# Oleifolioside A, a New Active Compound, Attenuates LPS-Stimulated iNOS and COX-2 Expression through the Downregulation of NF-**κ**B and MAPK Activities in RAW 264.7 Macrophages

**DOI:** 10.1155/2012/637512

**Published:** 2012-07-17

**Authors:** Hai Yang Yu, Kyoung-Sook Kim, Young-Choon Lee, Hyung-In Moon, Jai-Heon Lee

**Affiliations:** College of Natural Resources and Life Science, BK21 Center for Silver-Bio Industrialization, Dong-A University, Busan 604-714, Republic of Korea

## Abstract

Oleifolioside A, a new triterpenoid compound isolated from *Dendropanax morbifera* Leveille (*D. morbifera*), was shown in this study to have potent inhibitory effects on lipopolysaccharide (LPS-)stimulated nitric oxide (NO) and prostaglandin E_2_ (PGE_2_) production in RAW 264.7 macrophages. Consistent with these findings, oleifolioside A was further shown to suppress the expression of LPS-stimulated inducible nitric oxide synthase (iNOS) and cyclooxigenase-2 (COX-2) in a dose-dependent manner at both the protein and mRNA levels and to significantly inhibit the DNA-binding activity and transcriptional activity of NF-*κ*B in response to LPS. These results were found to be associated with the inhibition of the degradation and phosphorylation of I*κ*B-*α* and subsequent translocation of the NF-*κ*B p65 subunit to the nucleus. Inhibition of NF-*κ*B activation by oleifolioside A was also shown to be mediated through the prevention of p38 MAPK and ERK1/2 phosphorylation. Taken together, our results suggest that oleifolioside A has the potential to be a novel anti-inflammatory agent capable of targeting both the NF-*κ*B and MAPK signaling pathways.

## 1. Introduction

Dendropanax* morbifera* Leveille (*D. morbifera*), which is distributed throughout the southern part of Korea, is an economically important tree species because of its role in the production of golden varnishes. The roots and stems of this plant have also been commonly used in traditional medicine for the treatment of several diseases, including headache, infectious diseases, skin diseases, and malady [1]. Previous studies have shown that *D. morbifera* extracts have anticomplement [2, 3], antiatherogenic [4], antiplasmodial [5], and antidiabetic [6] activities *in vitro* or *in vivo*. In this study, we isolated oleifolioside A, a cycloartane-type triterpene glycoside with diverse biological activities, from the lower stem parts of *D. morbifera* [5]. Although oleifolioside A has previously been identified in *Astragalus oleifolius* [7], its physiological activities have not been studied to date on anti-inflammatory action. We have demonstrated here for the first time that oleifolioside A has potent anti-inflammatory activity because of its inhibition of lipopolysaccharide (LPS-) stimulated nitric oxide (NO) and prostaglandin E_2_ (PGE_2_) expression in RAW 264.7 macrophages.

Inflammation is an important process in the immune response initiated by immune cells toward various pathogens and tissue injuries [8] and proinflammatory cytokines activate immune cells to upregulate inflammation [9]. During the inflammation process, macrophages play an important role in defensing against injurious agent prior to leukocyte migration. Activated macrophages produce further proinflammatory mediators, such as, NO and PGE_2_ [10]. NO, which plays a key role in disease pathophysiology, is a free radical produced from L-arginine after the stimulation of inducible nitric oxide synthase (iNOS) [11], while PGE_2_ is a prostaglandin produced from arachidonic acid by cyclooxigenase-2 (COX-2) that, in association with inflammatory mediators, plays an important role in inflammation. It is well known that the overproduction of these proinflammatory mediators contributes to the development and courses of various diseases, such as, rheumatoid arthritis, autoimmune disorders, neurodegenerative disease, and cancer [12–15].

In the current study, we investigated the anti-inflammatory mechanisms by which oleifolioside A inhibits the production of proinflammatory mediators in LPS-stimulated RAW 264.7 macrophages.

## 2. Materials and Methods

### 2.1. Materials

LPS, 3-(4,5-dimethylthiazol-2-yl)-2,5-diphenyl-tetrazolium bromide (MTT) L-N^6^-(1-Iminoethyl)lysine hydrochloride(NIL) and NS-398 were purchased from Sigma Chemical (St. Louise, MO, USA). Antibodies specific for iNOS, COX-2, p50, p65, I*κ*B-*α*, p-I*κ*B-*α*, and goat anti-rabbit IgG-FITC were obtained from Santa Cruz Biotechnology (CA, USA), while antibodies specific for MAPK family proteins (ERK1/2, JNK, and p38 MAPK) and phospho-MAPK family proteins were purchased from Cell Signaling (Beverly, MA, USA). Peroxidase-labeled donkey anti-rabbit, sheep anti-mouse immunoglobulin, and an enhanced chemiluminescence (ECL) kit were purchased from GE Healthcare Bio-Sciences Corp (Piscataway, NJ,USA). Oleifolioside A isolated from *D. morbifera* was prepared as described previously [7], dissolved in dimethyl sulfoxide (DMSO) as a stock solution at 30 mM concentration, and stored in aliquots at −20°C.

### 2.2. Cell Culture and Cell Viability Assay

RAW 264.7 macrophages were obtained from the American Type Culture Collection and cultured in DMEM (Gibco BRL, Gaithersburg, MD, USA) supplemented with 10% heat-inactivated FBS, 100 U/mL penicillin, and 100 **μ**g/mL streptomycin (Gibco BRL, Gaithersburg, MD, USA) in a humidified incubator with 5% CO_2_ at 37°C. An MTT assay was used to determine cell viability.

### 2.3. Measurement of NO Levels

As nitrite is a major stable product of NO, the concentration of NO in culture supernatants was determined by measuring the nitrite levels by using Griess reagent (St. Louise, MO, USA). Cells were stimulated with LPS in 6-well plates for 24 h, and then 100 **μ**L of each culture medium was mixed with an equal volume of Griess reagent. Nitrite levels were determined using an ELISA plate reader at 540 nm, and concentrations were calculated by reference to a standard curve generated from known concentrations of sodium nitrite.

### 2.4. Measurement of PGE_2_ Levels

RAW 264.7 macrophages were subcultured in 6-well plates and pretreated with the indicated concentrations of oleifolioside A in the presence or absence of LPS (500 ng/mL) for 24 h. The PGE_2_ concentration was determined using 100 **μ**L of the culture medium supernatant in an enzyme immunosolvent assay ELISA (Cayman, MI, USA).

### 2.5. RNA Isolation and RT-PCR

Total RNA was extracted from cells by using Trizol reagent (Invitrogen, Carlsbad, CA, USA), following the manufacturer's protocol. Reverse-transcription polymerase chain reaction (RT-PCR) was then performed according to the manufacturer's instructions to synthesize cDNA from 1 **μ**g of total RNA by using the AMV RNA PCR Kit (Takara, Japan). The iNOS and COX-2 genes, as well as the *β*-actin gene as an internal control, were subsequently amplified from the cDNA by PCR using previously described primer sequences [16]. PCR products were electrophoresed in a 1% agarose gel and visualized following staining with ethidium bromide.

### 2.6. Western Blot Analysis

After treatment with various concentrations of oleifolioside A in the presence or absence of 500 ng/mL LPS, cells were analyzed by immunoblotting as described previously [16]. Primary antibodies that recognize iNOS, COX-2, and the phospho- or total forms of ERK1/2, JNK, p38 MAPK, p50, and p65 were used, and specific proteins were detected using an enhanced chemiluminescence kit (GE Healthcare Bio-Sciences Corp,Piscataway, NJ,USA). Equal loading was assessed using GAPDH and hnRNP antibodies (Chemicon, El Segundo, CA, USA), and the amounts of total protein were normalized to the levels of these proteins.

### 2.7. Electrophoretic Mobility Shift Assay

Nuclear extracts were prepared as described previously [17]. To determine the level of NF-*κ*B activation, an EMSA was performed using a gel shift assay system kit (Promega, Madison, WI, USA) according to the manufacturer's instructions. Briefly, EMSA probes were created by end-labeling double-stranded oligonucleotides containing the consensus sequence for NF-*κ*B (5′-CCAGTGGAATTCCCCAG-3′) (Promega, Madison, WI, USA) with [*γ* ^32^-P] ATP (3000 Ci/mmol; GE Healthcare Bio-Sciences Corp,Piscataway, NJ,USA) by using T4 polynucleotide kinase (Takara, Japan). Competition (C.P.) was performed using a 100-fold excess of unlabeled NF-*κ*B consensus oligonucleotides. DNA-protein complexes were separated from the unbound DNA probe in a 4% nondenaturing polyacrylamide gel electrophoresed in 0.5× TBE running buffer at 300 V for 15 min. The gel was subsequently vacuum-dried and exposed to an X-ray film overnight.

### 2.8. Transient Transfection and Luciferase Reporter Assay

NF-*κ*B reporter constructs were purchased from Clontech (Palo Alto, CA, USA). Cells were cultured and grown overnight before being cotransfected with 1 **μ**g of various plasmid constructs and 0.5 **μ**g of the pCMV-*β*-galactosidase reporter plasmid. Transfection was performed for 5 h by using Lipofectamine reagent (Invitrogen, San Diego, CA, USA). After transfection, the cells were starved in serum-free media for 6 h before being exchanged with fresh serum-free media containing a range of oleifolioside A concentrations (0–15 **μ**M) in the presence of LPS (500 ng/mL). Following further 24 h incubation, cells were lysed in 200 **μ**L of RIPA buffer (Pierce, Rockford, IL, USA), and luciferase activity was assayed using the dual luciferase report assay system (Promega, Madison, WI, USA) and luminometer (Promega, Madison, WI, USA).

### 2.9. Immunofluorescence Analysis

Cells were cultured directly on glass cover slips in a 35 mm dish for 24 h and then pretreated with oleifolioside A for 1 h 500 ng/mL of LPS, which were either pretreated or not pretreated with. Cells were fixed with 3.7% paraformaldehyde, treated with 0.2% triton X-100, and blocked with 2% BSA. To investigate the cellular localization of NF-*κ*B, cells were incubated with a polyclonal antibody (1 : 100) against NF-*κ*B p65 for 1 h. After extensive washing with PBS, cells were further incubated with a secondary FITC-conjugated donkey anti-rabbit IgG antibody (1 : 100) for 1 h at room temperature. Nuclei were stained with DAPI solution (1 **μ**g/mL), and the cells were analyzed by confocal microscopy using a fluorescence microscope (Carl Zeiss, Germany).

### 2.10. Flow Cytometric Analysis

Following treatment with the indicated concentrations of oleifoliosides A in the presence or absence of LPS (500 ng/mL) for 24 h, cells were collected, washed with cold PBS, and fixed in 75% ethanol at 4°C for 30 min. The DNA content of the cells was measured using a DNA staining kit (CycleTEST PLUS Kit, Becton Dickinson, San Jose, CA, USA). Propidium iodide (PI-) stained nuclear fractions were obtained by following the kit protocol. The cells were then filtered through 35 mm mesh, and DNA content fluorescence was determined using a FACS Caliber (Becton Dickinson) flow cytometer within 1 h. The cellular DNA content was analyzed by CellQuest software (Becton Dickinson).

### 2.11. Statistical Analysis

Data are expressed as the mean ± S.E. for the number of experiments. All experiments (*n* = 6) were conducted. Statistical analyses were conducted using Sigmaplot software (version 11.0). Comparisons between the two groups were analyzed using the Student's *t*-test. *P < 0.05 and **P < 0.01 were considered statistically significant.

## 3. Results

### 3.1. Inhibitory Effects of Oleifolioside A on Cell Viability and NO and PGE_2_ Production in LPS-Stimulated RAW 264.7 Macrophages

**Figure 1:**
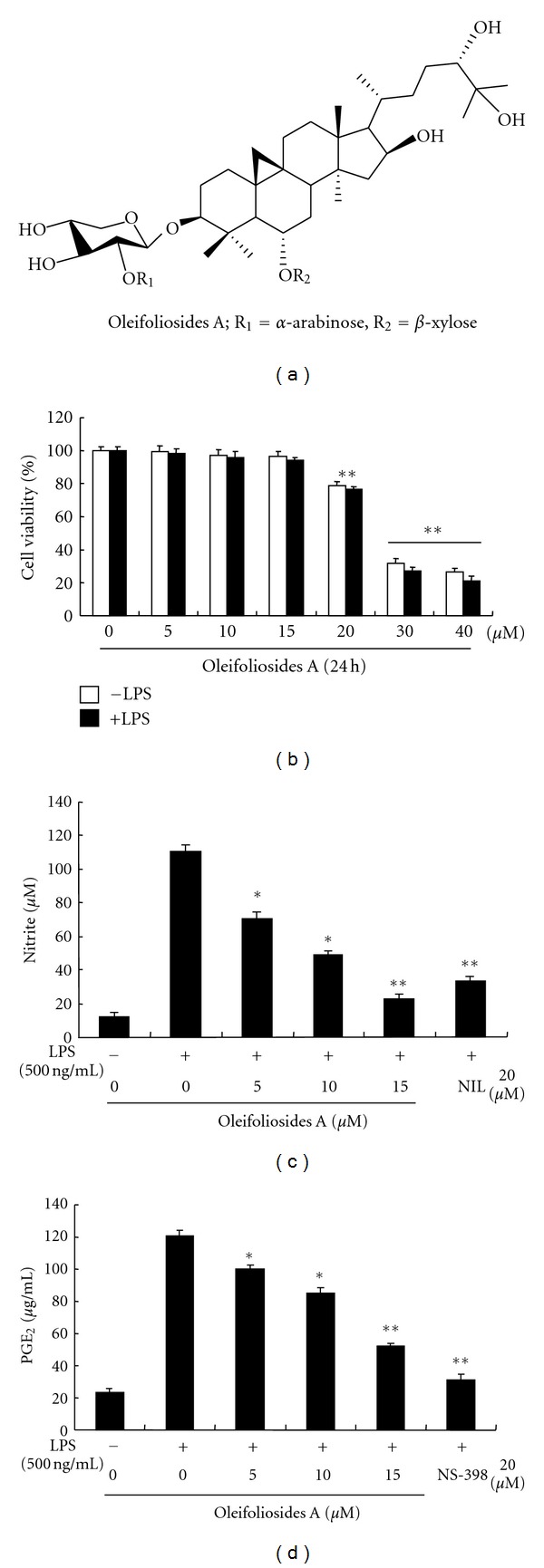
Effects of oleifoliosides A on LPS-stimulated cell viability and NO, PGE_2_ production in RAW 264.7 macrophages. (a) Chemical structure of oleifoliosides A. (b) Cells were treated with the concentrations of oleifoliosides A (0, 5, 10, 15, 20, 30, or 40 **μ**M) 1 h before LPS (500 ng/mL) presence or absence for 24 h. Cell viability was assessed by MTT assays. (c) Cells were treated with the indicated concentrations of oleifoliosides A (0, 5, 10, or 15 **μ**M) and NIL (20 **μ**M) 1 h before LPS (500 ng/mL) treatment for 24 h, the nitrite production was measured by the Griess reaction. (d) Cells were treated with the indicated concentrations of oleifoliosides A and NS-398 (20 **μ**M) 1 h before LPS (500 ng/mL) treatment for 24 h, the PGE_2_ concentration was determined by ELISA kit. The values are presented as mean ± S.E. of three independent experiments. *n* = 6 per experiment. *P < 0.05 versus LPS and*P < 0.01 versus LPS.

To investigate the effects of oleifolioside A on cell viability, RAW 264.7 macrophages were treated with LPS in the presence or absence of oleifolioside A, and cell numbers were assessed by MTT assays. As oleifolioside A did not significantly affect cell viability, even at a concentration of 15 **μ**M (Figure 1(b)), these results can be further identified by flow cytometric analysis (Figure S1) (see Supplementary Material available online at doi:10.1155/2012/637512). So concentrations ranging from 0 to 15 **μ**M were used in subsequent experiments.

The potential anti-inflammatory effects of oleifolioside A on LPS-stimulated NO and PGE_2_ production were examined in RAW 264.7 macrophages by pretreating cells with various concentrations of oleifolioside A for 1 hbefore stimulation with 500 ng/mL LPS for 24 h. NO and PGE_2_ concentrations in the culture medium were measured by using Griess reagent and ELISA, respectively. As shown in Figures 1(c) and 1(d), respectively, NO and PGE_2_ production was markedly induced in LPS-stimulated RAW 264.7 macrophages when compared to unstimulated negative controls, while pretreatment with oleifolioside A significantly prevented this increase, in a dose-dependent manner. Positive control cells treated with NIL (20 **μ**M), a selective effective inhibitor of inducible nitric oxide synthase (iNOS) induced by LPS [18], and NS-398 (20 **μ**M), a selective inhibitor of cyclooxygenase (COX)-2 [19], also exhibited markedly reduced NO and PGE_2_ levels in LPS-stimulated RAW 264.7 macrophages. These results suggest that the inhibition of production of NO and PGE_2_ in LPS-stimulated RAW 264.7 macrophages is not due to oleifolioside A cytotoxicity.

### 3.2. Inhibitory Effects of Oleifolioside A on LPS-Stimulated iNOS and COX-2 Expression

**Figure 2:**
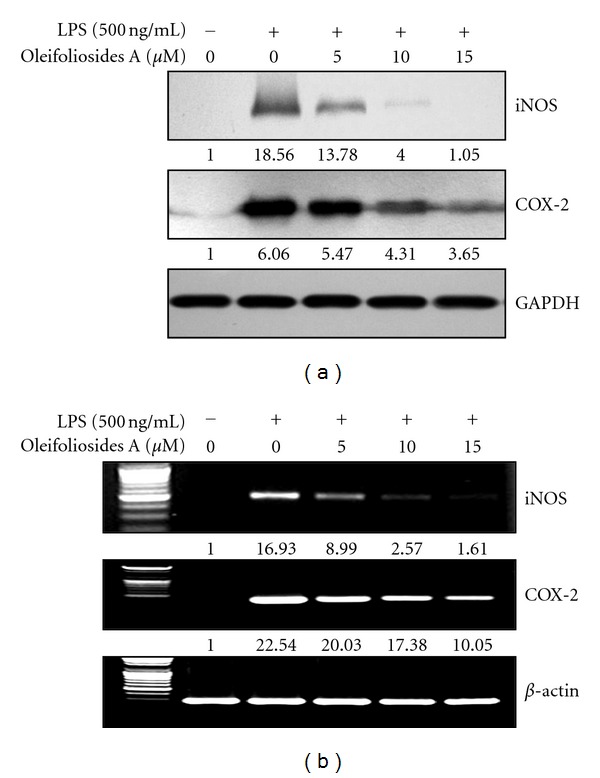
Effects of oleifoliosides A on LPS-stimulated iNOS and COX-2 expression. (a) Cells were pretreated with the indicated concentrations of oleifoliosides A 1 h before LPS (500 ng/mL) treatment for 24 h. The cell lysates were used to determine iNOS and COX-2 protein levels by Western blot assay. (b) After LPS treatment for 6 h, the levels of iNOS and COX-2 mRNA were determined by RT-PCR. GAPDH and *β*-actin were used as internal controls for Western blot and RT-PCR assays, respectively, *n* = 6 per experiment.

In an attempt to assess whether the inhibition of NO and PGE_2_ production by oleifolioside A was related to the downregulation of iNOS and COX-2, we next examined the expression levels of these enzymes by Western blotting and quantitative RT-PCR. As shown in Figure 2(a), both iNOS and COX-2 protein levels greatly increased in LPS-stimulated cells when compared to the controls. However, this induction was significantly inhibited in a dose-dependent manner by treatment with oleifolioside A. In particular, the level of iNOS protein was almost completely suppressed when cells were treated with 15 **μ**M oleifolioside A. Similarly, when the effect of oleifolioside A treatment on iNOS and COX-2 mRNA expression in LPS-stimulated RAW 264.7 macrophages was examined by quantitative RT-PCR, a corresponding decrease in iNOSandCOX-2mRNAs was observed (Figure 2(b)). These results suggest that the inhibitory effects of oleifolioside A on LPS-stimulated iNOS and COX-2 expression levels were responsible for the inhibition of NO and PGE_2_ production observed in these cells.

### 3.3. Inhibitory Effects of Oleifolioside A on LPS-Stimulated Phosphorylation of I*κ*B-*α* and p65 Nuclear Translocation

**Figure 3:**
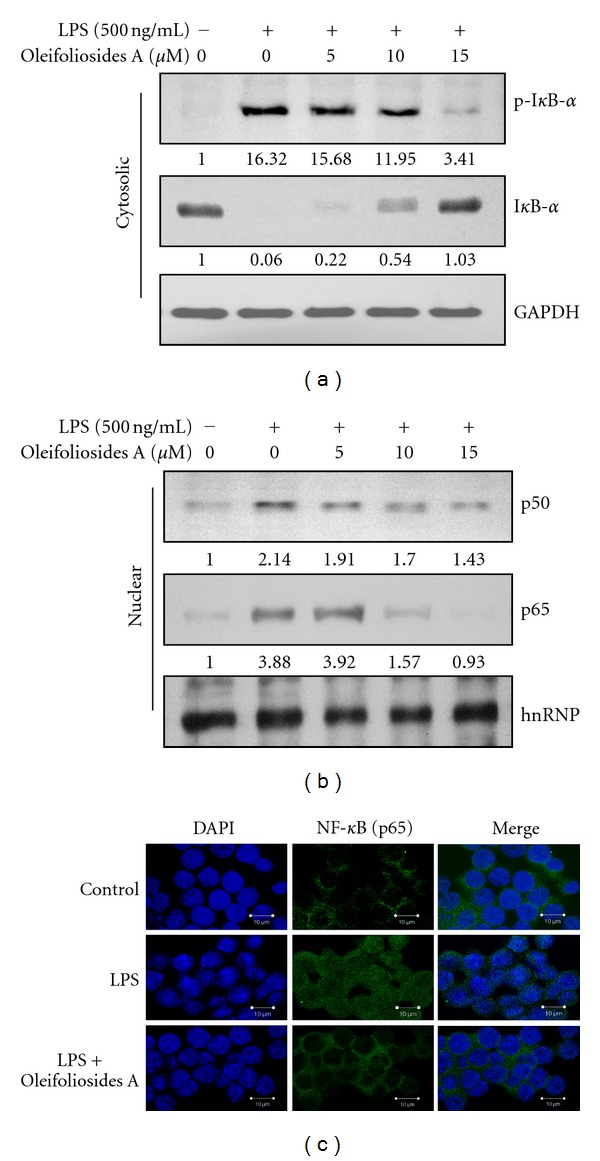
Effects of oleifoliosides A on LPS-stimulated phosphorylation and degradation of I*κ*B-*α* and nuclear translocation of p65. (a) and (b) Cells were pretreated with the indicated concentrations of oleifoliosides A 1 h and then LPS (500 ng/mL) treatment for 15 or 60 min. Cytosol or nuclear protein was determined by Western blot assay using anti-p-I*κ*B-*α*, I*κ*B-*α*, NF-*κ*B p50, and NF-*κ*B p65. GAPDH and hnRNP were used as internal controls for Western blot. (c) The cells were pretreated with 15 **μ**M oleifoliosides A for 1 h prior to stimulation with LPS (500 ng/mL) for 1 h. The nuclear localization of NF-*κ*B p65 was determined using confocal laser scanning microscopy after being stained with DAPI, anti-NF-*κ*B p65, and FITC-labeled anti-rabbit IgG antibody. *n* = 6 per experiment.

NF-*κ*B is sequestered into an inactive cytoplasmic complex by binding to an inhibitory *κ*B protein, I*κ*B. Exposure cells to external stimuli, such as, LPS, can cause rapid phosphorylation and subsequent degradation of I*κ*B. Meanwhile, NF-*κ*B is released and activated. During this process, I*κ*B is dissociated from NF-*κ*B, and the I*κ*B content in the cytosol reflects the condition of NF-*κ*B activation. In the present study, we examined the effect of oleifoliosides A on the LPS-stimulated phosphorylation and degradation of I*κ*B-*α* by Western blotting. As shown in Figure 3(a), thephosphorylation of I*κ*B-*α* after LPS treatment was dramatically inhibited by oleifolioside A in a dose-dependent manner. Furthermore, I*κ*B-*α* was markedly degraded after treatment with LPS, whereas treatment with oleifolioside A prevented this degradation in a dose-dependent manner. Since the dissociation of I*κ*B-*α* from NF-*κ*B allows the activated free dimer subunits of NF-*κ*B (p50/p65) to translocate into the nucleus from the cytosol [20], we examined whether oleifolioside A is capable of preventing this translocation by Western blotting. As shown in Figure 3(b), the treatment of RAW 264.7 macrophages with LPS alone markedly increased the amounts of NF-*κ*B p50 and p65 subunits in the nuclear fraction, which was prevented by pretreatment with oleifolioside A. GAPDH and hnRNP were used as internal controls for cytosol and nuclear proteins, respectively (Figures 3(a) and 3(b)). To clearly prove the influence of oleifolioside A on NF-*κ*B p65 nuclear translocation, the shift of NF-*κ*B to the nucleus in RAW 264.7 macrophages was also analyzed using immunofluorescence staining and visualization with confocal microscopy (Figure 3(c)). The confocal images revealed that while NF-*κ*B p65 was normally sequestered in the cytoplasm, the nuclear accumulation of NF-*κ*B p65 was strongly induced after LPS stimulation, and the LPS-stimulated translocation of NF-*κ*B p65 was effectively abolished after pretreatment with oleifolioside A. Taken together, these data suggest that the anti-inflammatory effect of oleifolioside A in LPS-stimulated RAW 264.7 macrophages involves the NF-*κ*B pathway.

### 3.4. Inhibitory Effects of Oleifolioside A on LPS-Stimulated NF-*κ*B DNA-Binding and Transcriptional Activity

**Figure 4:**
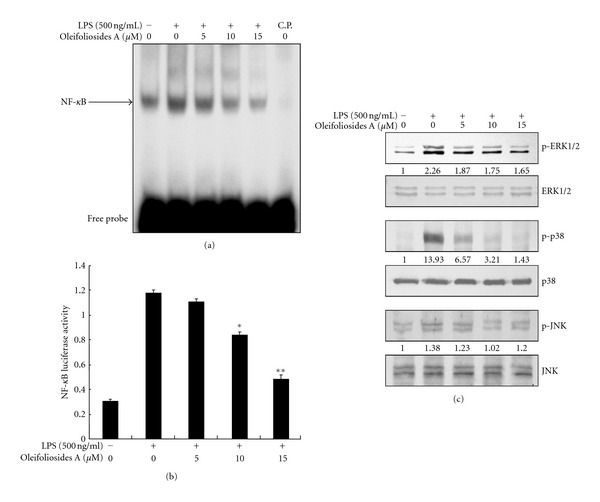
Effects of oleifoliosides A on LPS-stimulated NF-*κ*B activation and phosphorylation of MAPK signaling pathway. (a) Cells were pretreated with the indicated the concentrations of oleifoliosides A for 1 h and then stimulated with LPS (500 ng/mL) for 1 h. Nuclear extracts were prepared and analyzed for NF-*κ*B DNA-binding activity by EMSA. The C.P. represents 100-fold cold probe only. (b) Cells were transiently cotransfected with pNF-*κ*B-Luc reporter plasmid and then were pretreated with the indicated the concentrations of oleifoliosides A for 1 h. LPS (500 ng/mL) was then added and cells were further incubated 24 h. The cells were harvested and then the luciferase activities were determined by using the dual luciferase report assay system. (c) Cells were pretreated with the indicated concentrations of oleifoliosides A for 1 h before LPS (500 ng/mL) treatment for 15 min. and the levels of total/phospho-ERK1/2, JNK, and p38 MAPK were determined by Western blotting. The values are presented as mean±S.E. of three independent experiments. *n* = 6 per experiment, *P < 0.05 versus LPS and **P < 0.01 versus LPS.

NF-*κ*B is a key transcription factor involved in general inflammation as well as immune responses. Its activation is critically required for the expression of proteins, including iNOS and COX-2, in macrophages. To evaluate the molecular mechanism through which oleifolioside A inhibits the expression of such proinflammatory mediators, we examined NF-*κ*B DNA-binding activity by EMSA. As shown in Figure 4(a), treatment with LPS alone caused a marked increase in the DNA-binding activity of NF-*κ*B. In contrast, pretreatment with oleifolioside A significantly attenuated LPS-stimulated NF-*κ*B DNA-binding activity in a dose-dependent manner. To confirm the results of EMSA, we also investigated the effects of oleifolioside A on NF-*κ*B-dependent reporter gene expression following LPS treatment by luciferase reporter assay. RAW 264.7 macrophages were transiently cotransfected with a pNF-*κ*B-leu reporter vector generated by inserting four spaced NF-*κ*B-binding sites into the pLuc-promoter vector and then stimulated with 500 ng/mL LPS with or without oleifolioside A. As shown in Figure 4(b), treatment with oleifolioside A significantly reduced the level of NF-*κ*B luciferase activity stimulated by LPS in a dose-dependent manner. Taken together, these findings demonstrate that oleifolioside A suppresses the expression of iNOS and COX-2, at least in part via an NF-*κ*B-dependent mechanism.

### 3.5. Inhibitory Effects of Oleifolioside A on the LPS-Stimulated Phosphorylation of MAPKs

MAPK proteins play a critical role in the induction of proinflammatory mediators, such as, iNOS and COX-2, as well as in the activation of transcription factors, such as, NF-*κ*B [16]. Thus, MAPK proteins provide specific targets for inflammatory responses. To confirm whether the inhibition of NF-*κ*B activation by oleifolioside A is mediated through the MAPK pathways, we examined the effect of oleifolioside A on the LPS-stimulated phosphorylation of ERK1/2, JNK, and p38 MAPK in RAW 264.7 cells by Western blotting. As shown in Figure 4(c), LPS markedly induced the phosphorylation of ERK1/2, JNK, and p38 MAPK. Interestingly, while pretreatment with oleifolioside A it significantly inhibited the LPS-stimulated phosphorylation of p38 MAPK, slightly attenuated the phosphorylation of ERK1/2, and did not affect LPS-stimulated JNK phosphorylation. This suggests that ERK1/2 and p38 MAPK, but not JNK, are involved in the inhibitory effect of oleifolioside A on LPS-stimulated expression of proinflammatory mediators, such as, NO and PGE_2_.

## 4. Discussion

In an attempt to search for bioactive natural products capable of exerting potent physiological activities, we found, for the first time, that oleifolioside A, a cycloartane-type glycoside, isolated from the lower stem of *D. morbifera* has potent anti-inflammatory activity. We further showed that this effect is a result of the ability of oleifolioside A to prevent the increases in the levels of NO and PGE_2_ that occur in RAW 264.7 macrophages following LPS stimulation. NO, a short-lived free radical, is an important regulatory molecule in various physiological and pathological processes, including cancer, cardiovascular diseases, Alzheimer's disease, neurologic diseases, and [21–23]. Overproduction of NO by iNOS through oxidative deamination of L-arginine in response to proinflammatory cytokines and bacterial LPS is correlated with many types of inflammatory diseases [24]. PGE_2_, another potent inflammatory mediator produced from arachidonic acid by COX-2, enhances vascular permeability, resulting in fever, edema, and pain at the sites of inflammation. While COX-2 expression is barely detectable under normal physiological conditions, it is rapidly and transiently induced by proinflammatory mediators and mitogenic stimuli, such as, cytokines, phorbol ester, and LPS [25]. Thus, the inhibition of iNOS and COX-2 expression may prove to be a key therapeutic target for the treatment of inflammatory diseases. In this study, oleifolioside A significantly inhibited LPS-stimulated NO and PGE_2_ production in a dose-dependent manner (Figures 1(c) and 1(d)). Consistent with these findings, oleifolioside A also suppressed LPS-induced expression of iNOS and COX-2 at the protein and mRNA levels in RAW 264.7 macrophages (Figures 2(a) and 2(b)), suggesting that the observed reductions in NO and PGE_2_ release following treatment with oleifolioside A may have been due to the transduction and transcriptional suppression of iNOS and COX-2 genes.

Activation of NF-*κ*B, a nuclear transcription factor, is pivotal for the expression of genes encoding proinflammatory mediators, including NO, PGs, and inflammation-related protein [26]. It is well known that the suppression of NF-*κ*B transcriptional activity can inhibit the expression of many proinflammatory genes in cells induced by LPS [17]. In unstimulated cells, NF-*κ*B exists as homodimeric or heterodimeric complexes of p50 and p65 subunits bound to I*κ*B and remains inactive in the cytoplasm of cells. NF-*κ*B activation by stimuli, such as, LPS results in the phosphorylation, ubiquitination, and proteasome-mediated degradation of I*κ*B protein, leading to the release of NF-*κ*B and its translocation from the cytoplasm to the nucleus. It then binds DNA at *κ*B-binding motifs and promotes the expression of inflammation-related genes [27, 28].

The present study shows that oleifolioside A markedly inhibits rapid phosphorylation of I*κ*B-*α* with subsequent degradation by proteasomes in the cytosol (Figure 3(a)), as well as prevents the translocation of the p65 subunit of NF-*κ*B from the cytosol to the nucleus (Figures 3(b) and 3(c)). We also found that oleifolioside A significantly inhibited NF-*κ*B activity in LPS-stimulated RAW 264.7 macrophages (Figures 4(a) and 4(b)). Our data indicate that oleifolioside A may inhibit LPS-stimulated NF-*κ*B activation through the suppression of the phosphorylation and degradation of I*κ*B-*α* and subsequent effects on the nuclear translocation of the p65 subunit of NF-*κ*B in RAW 264.7 macrophages. 

MAPK proteins play a critical role in the induction of proinflammatory mediators by regulating the activation of NF-*κ*B [16, 17, 29]. Previous studies have shown that all three MAPK cascades [16], ERK1/2 [29], and both ERK1/2 and p38 MAPK pathways [29] are involved in LPS-stimulated iNOS expression and inflammatory signaling in RAW 264.7 macrophages. Thus, we investigated the effect of oleifolioside A on the LPS-stimulated phosphorylation of MAPK in RAW 264.7 macrophages. Interestingly, only the phosphorylation of p38 MAPK and ERK1/2 in response to LPS was decreased by oleifolioside A treatment. However, oleifolioside A was not observed to have any effect on the LPS-stimulated phosphorylation of JNK (Figure 4(c)). Recently, involvement of the phosphoinositide 3-kinase (PI3 K)/Akt pathway in expression of inflammatory mediators in RAW 264.7 macrophages through activation of NF-*κ*B has been demonstrated [30]. However, in the present study, regulation of the Akt pathway was not examined in oleifoliosides-A-treated macrophages (data not shown). These results suggest that the suppression of p38 MAPK and ERK1/2 phosphorylation by oleifolioside A may be involved in the inhibition of proinflammatory mediators following LPS-stimulated NF-*κ*B activation.

In conclusion, oleifolioside A was shown to inhibit the production of proinflammatory mediators [NO, PGE_2_] induced by LPS as well as their expression levels [iNOS, COX-2], through the inhibition of I*κ*B-*α* phosphorylation and p65 nuclear translocation in LPS-stimulated RAW 264.7 macrophages. Our data indicate that oleifolioside A resembles to an anti-inflammatory molecule by suppressing of NF-*κ*B activation via downregulating the p38 MAPK and ERK1/2 phosphorylation in RAW 264.7 macrophages. Considering these results, oleifolioside A may be a novel anti-inflammatory agent that could be used in the medication of inflammation-related diseases. Further research will be required to evaluate the potential *in vivo* anti-inflammatory effects of oleifolioside A.

## Supplementary Material

Figure S1: Effects of oleifolioside A on LPS-stimulated cell viability by flow cytometric analysis.
