# Supplementary figures and images for: Oleifolioside A, a New Active Compound, Attenuates LPS-Stimulated iNOS and COX-2 Expression through the Downregulation of NF-κB and MAPK Activities in RAW 264.7 Macrophages
Source: Evid Based Complement Alternat Med. 2012 Jul 17;2012:637512. doi: 10.1155/2012/637512 (PMC3405816; doi:10.1155/2012/637512)

***
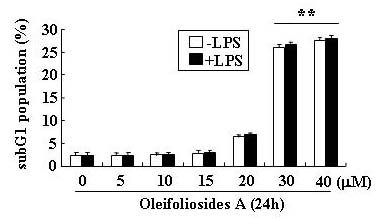
***

**FIGURE S1. Yu *et al.***

Supplement: Supplementary file 1 — Figure S1: Effects of oleifolioside A on LPS-stimulated cell viability by flow cytometric analysis. [file 637512.f1.docx]
